# Protease-Activated Receptor 2 in inflammatory skin disease: current evidence and future perspectives

**DOI:** 10.3389/fimmu.2024.1448952

**Published:** 2024-09-05

**Authors:** Mengjie Fan, Xiaoyao Fan, Yangfan Lai, Jin Chen, Yifan Peng, Yao Peng, Leihong Xiang, Ying Ma

**Affiliations:** ^1^ Department of Dermatology, Huashan Hosptial, Fudan University, Shanghai, China; ^2^ iHuman Institute, ShanghaiTech University, Shanghai, China

**Keywords:** Protease-Activated Receptor 2, inflammatory skin disease, serine protease, skin barrier, acne vulgaris, atopic dermatitis, targeted therapy

## Abstract

Protease-activated receptor-2 (PAR2) is a class-A G protein-coupled receptor (GPCR) activated by serine proteases and is expressed by multiple tissues, including the skin. PAR2 is involved in the skin inflammatory response, promoting Th2 inflammation, delaying skin barrier repair, and affecting the differentiation of keratinocytes. It also participates in the transmission of itch and pain sensations in the skin. Increasing evidence indicates that PAR2 plays an important role in the pathogenesis of inflammatory skin diseases such as acne vulgaris, rosacea, psoriasis, and atopic dermatitis. Additional focus will be placed on potential targeted therapies based on PAR2. The Goal of this review is to outline the emerging effects of PAR2 activation in inflammatory skin disease and highlight the promise of PAR2 modulators.

## 1 Introduction

In 1994, Nystedt and colleagues first isolated a DNA sequence encoding a G-protein-coupled receptor (GPCR) from a mouse genomic library. The predicted protein is similar in structure to the thrombin receptor and has a similar activation mechanism. Because this was the second GPCR found to be activated by proteases, it was named Protease-Activated Receptor 2 (PAR2) (1). PAR2 has been primarily localized to the stratum granulosum layer of epidermal keratinocytes in healthy epidermis in adult humans. PAR2 can be activated by a variety of endogenous and exogenous proteases and act through several molecular pathways such as nuclear factor kappa B (NF‐κB), mitogen‐activated protein kinase (MAPK/ERK), transient receptor potential (TRP) channels and β‐arrestins (2, 3). Inflammation is the most prevalent pathological process in dermatological diseases, and the function of PAR2 in inflammation is well-established. PAR2 play important roles in regulating skin homeostasis, immune and inflammatory responses, and tumor surveillance (1, 2, 4, 5).

This review aims to delineate the emerging effects of PAR2 activation in inflammatory skin diseases, including psoriasis, atopic dermatitis, acne vulgaris, and systemic sclerosis (**Table 1**). The objective of this study is to provide a comprehensive overview of the current state of knowledge regarding PAR2 as a regulator of the immune response, inflammatory response, keratinocyte differentiation, and epidermal barrier. Additional focus will be placed on PAR2-targeted therapy in the field of inflammatory skin diseases; this publication is not intended to be an exhaustive review of all emerging PAR2 modulators.

**Table 1:** Summary of effects of activation of PAR2 on inflammatory skin diseases.

Representative diseases	Potential evidence	References
**Acne vulgaris**	1. Increased protease activity at lesion sites. 2. *C. acnes* culture supernatant activates PAR2 in keratinocytes, upregulating AMPs and MMPs. 3. *C. acnes* culture supernatant increased the lipid production of SZ95 sebocytes via PAR2. 4. Tetracyclines and macrolides relieve acne symptoms by regulating the PAR-2/IL-8 axis.	(25, 31, 52–55)
**Rosacea**	1. Higher serine protease activity and LL-37 expression in rosacea skin compared to normal skin. 2. Positive correlation between LL-37 and PAR2 expression in immunohistochemical staining	(58, 60–62)
**Psoriasis**	1. Increased PAR2 expression in psoriasis patients. 2. HAT may upregulate IL-8 via PAR2 activation. 3. Monocytes/macrophages from PsA patients increase MCP-1 levels in response to PAR2 activation.	(64–68)
**Atopic dermatitis**	1. Elevated epidermal serine protease activity in AD patients. 2. Detection of KLK5, -7, -8, and -14 in the perspiration of AD patients 3. Murine models overexpressing PAR2 develop AD-like symptoms. 4. PAR2 overexpression in keratinocytes and immune cells induces IL-6, ICAM-1, and TSLP. 5. PAR2 signaling activates histamine-independent itching pathways. 6. PAR2 antagonist PZ235 downgrades inflammatory factors and alleviates AD symptoms.	(22, 24, 37, 51, 71–79)
**Systemic Sclerosis**	1. PAR2 immunoreactivity in fibroblasts of SSc skin, but not in healthy skin. 2. PAR2 co-localized with 21 ± 8% a-SMA-positive myofibroblasts in SSc skin. 3. PAR2-induced intracellular calcium mobilization in HDF after bleomycin pretreatment. 4. PAR2 silencing prevents sDPP4-induced ERK1/2 activation, NF-κB phosphorylation, and pro-inflammatory factors upregulation in human smooth muscle cells. 5. PAR2 antagonist or knocking down PAR2 can prevent sDPP4–induced fibrotic marker expression via SMAD/NF-κb signaling in fibroblasts.	(81–85)

*C. acnes*, Cutibacterium acnes; AMP, Antimicrobial Peptide; MMP, Matrix Metalloproteinase; HAT, Human airway trypsin-like protease; PsA, Psoriatic Arthritis; MCP-1, Monocyte Chemoattractant Protein-1; AD, atopic dermatitis; ICAM-1, Intercellular cell adhesion molecule-1; TSLP, Thymic stromal lymphopoietin; HDF, human dermal cultured fibroblasts; sDPP4, soluble Dipeptidyl peptidase-4.

### 1.1 Structure and signal transduction pathway of PAR2

The human PAR2 molecule is a transmembrane protein composed of 397 amino acids, consisting of extracellular regions (N-terminus and extracellular loops), transmembrane regions (seven transmembrane helices), and intracellular regions (intracellular loops and C-terminus). PAR2 is expressed on various structural cells including smooth muscle, epithelial, and endothelial cells (3). Activation of PAR2 is initiated by cleavage of the N terminus of the receptor by a serine protease resulting in the generation of a new tethered ligand that interacts with the receptor within extracellular loop-2. The intracellular C-terminus affects signal transduction, desensitization, and receptor internalization (6). Besides PAR2, the known PARs family includes PAR1-4. PAR1, 3, and 4 were originally considered thrombin receptors and mainly mediate thrombin signaling in the form of heterodimers (3). PAR2 can be irreversibly activated by serine proteases (SPs) such as trypsin, tryptase, and neutrophil elastase through the cleavage of the N-terminus at Arg35-Ser36, exposing a new N-terminal sequence (SLIGKV or SLIGKL). Then the N-terminal sequence folds over and self-activates PAR2 through binding to conserved regions of ECL2 (QTIFIPALNITTCHDVLPEOLLVG) (7). Synthetic peptides mimicking the new N-terminus of the cleaved receptor can also activate PAR2. SLIGKV-NH2, 2f-LIGRLI-NH2, GB110 and other PAR2 synthetic agonists, which have the same sequence as the tethered ligand domain of human PAR2, can simulate the activity of serine proteases.

**Figure 1:**
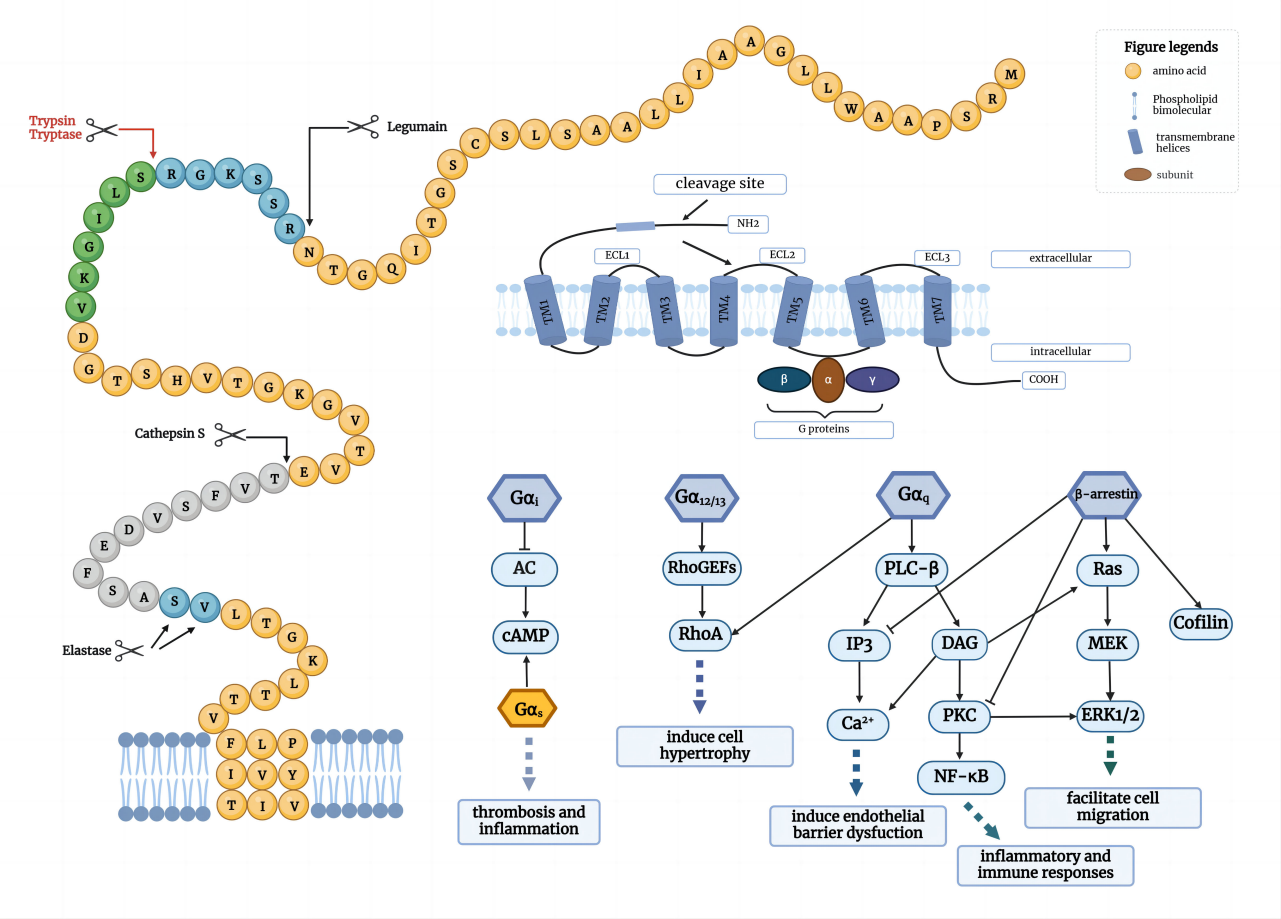
PAR2-mediated signal transduction. PAR2 is activated by several serine proteases and signal through Gα proteins (Gα_i_, Gα_12/13_ and Gα_q_) across different cell environments, as well as through G protein-independent proteins including β-arrestins. Gα_i_ can inhibit AC to regulate downstream cAMP, whereas Gα_s_ can activate cAMP; Gα_12/13_ can induce Rho-RhoGEFs to activate RhoA; Gα_q-_phospholipase C-β (PLC-β) can activate downstream IP3 or DAG, resulting in Ca^2+^ release and PKC activation; β-arrestin can activate ERK1/2 signaling pathway. These signal transductions can initiate some physiological changes, including cell hypertrophy, endothelial barrier dysfunction, cell migration and immune responses.

When activated by agonist peptides or proteases, PAR2 can trigger multiple signaling pathways in different cellular environments through classical G proteins (Gα_12/13_, Gα_q_, Gα_i_) or β-arrestin proteins, such as intracellular calcium ions [iCa^2+^], MAPK, ERK1/2, cAMP, PI3K, Rho kinase, and NF-κB pathways(**Figure 1**) (8–11). It is generally believed that after PAR2 activation, signal transduction occurs through a Gα_q-_ dependent pathway, activating phospholipase C (PLC) and producing inositol triphosphate (IP3) and diacylglycerol (DAG), further triggering the mobilization of Ca^2+^ and the activation of protein kinase C (PKC). Therefore, a common method to detect PAR2 activation is to measure intracellular Ca^2+^ flux (12). Downstream in this pathway, the phosphorylation of IKKα and IKKβ leads to the activation and nuclear translocation of NF-κB (13). NF-κB activation and induction of the inflammatory mediator intercellular adhesion molecule-1 (ICAM-1) may be a potential pro-inflammatory axis activated by PAR2 signaling (3). Research indicates that PAR2 is implicated in the development, prognosis, and occurrence of numerous inflammatory skin diseases. PAR2 can also regulate Rho-guanine nucleotide exchange factors (RhoGEFs) through Gα_12/13-_ dependent signaling and induce the activation of Rho-member A (RhoA), thereby regulating platelet secretion, gastrointestinal function, and cell differentiation. In the absence of G proteins, β-arrestins can function as scaffolding proteins in conjunction with PAR2, activating the ERK pathway and promoting the growth, invasion, and metastasis of tumor cells (14). β-arrestins are also significant negative regulators in GPCR signaling pathways, collaborating with G protein-coupled receptor kinases (GRKs) to facilitate desensitization (3). The proteolytic activation of PAR2 promotes GRK phosphorylation, providing binding sites for β-arrestins, thereby inducing the rapid translocation of β-arrestins from the cytoplasm to the cell membrane, forming PAR2/β-arrestins complexes, which subsequently bind to clathrin-coated vesicles for endocytosis (15, 16). In addition to modulating the coupling of receptors to signaling pathways, cells can also reduce responsiveness by decreasing the expression of PAR2 on the plasma membrane. After prolonged stimulation with agonists, cells degrade surface receptors after cleavage, thus reducing the total number of receptors and irreversibly terminating signal transduction (17). Consequently, the recruitment decouples Gα signaling but also facilitates receptor internalization and degradation. Consequently, PAR2 has the capacity to regulate intricate cellular processes by utilizing a variety of signaling pathways. This review seeks to outline the emerging effects of PAR2 activation in inflammatory skin diseases, especially its role in cell differentiation, barrier repair and inflammatory response. Additional focus will be placed on antagonist-, antibody- and pepducin-based modulators of PAR2, summarizing the development process of modulators targeting PAR2 in the field of inflammtory skin diseases.

## 2 PAR2 and skin

### 2.1 The pathological basis of PAR2 involvement in skin disease

#### 2.1.1 Expression and activation of PAR2 in skin

PAR2 is expressed in the granular layer of the epidermis, and on eosinophils, neutrophils, monocytes, macrophages, dendritic cells, mast cells, and T cells in the dermis (18–20). PAR2 can be activated by different endogenous or exogenous proteases, and its activation elicits changes in intracellular calcium levels. The main activators of PAR2 in the skin may be kallikreins (KLKs), serine protease channel-activating protein-1 (Cap1/Prss8), Cathepsin S, tryptase, and exogenous proteins such as dust mite and cockroach allergens (20). Stefansson et al. employed four human skin-derived KLKs to stimulate KNRK cells *in vitro* and conducted an intracellular calcium flux analysis to determine whether KLKs can cleave and activate PAR2. KLK5 and KLK14 were observed to activate PAR2, while KLK7 and KLK8 did not. KLK14 and PAR2 were also discovered to be co-expressed in inflammatory skin disease models, including rosacea and atopic dermatitis. This finding implies that KLKs may be endogenous PAR2 activators in keratinocytes and may be involved in the activation of PAR2 in the epidermis and the inflammatory response (21). Cathepsin S is a cysteine protease found in dendritic cells. It was further demonstrated to activate the inflammatory modulating agent PAR2 by cleaving its N-terminus-like serine proteases (22).

Both cockroach allergen extract and dust mite allergens can activate PAR2 through serine protease activity (23, 24). Kato et al. treat primary human keratinocytes with serine protease-rich extract of whole mite culture (WCE) and recombinant group 1 allergens (rDer f 1 and rDer p 1), which exclusively exhibit cysteine protease activity. They discovered that the protease activity of the WCE induced intracellular Ca^2+^ mobilization, whereas rDer f 1 and rDer p 1 did not. The results suggest that mite-derived serine protease activity may contribute to the pathogenesis of atopic dermatitis by activating keratinocytes via PAR2 activation (24). Jeong et al. further confirmed that allergens increased protease activities in the epidermis and delayed barrier recovery and lamellar body secretion in murine skin when applied topically to barrier-disrupted sites. Yet the barrier recovery was restored by the topical application of a PAR2 antagonist or protease inhibitors, indicating that cockroach allergens may be involved in skin barrier repair by activating PAR2 (23). The findings of Lee et al. demonstrated that supernatants from Cutibacterium acnes (*C. acnes*) cultures could activate PAR2 in keratinocytes, thereby upregulating the mRNA expression levels of inflammatory cytokines such as IL-1α, IL-8, and TNF-α (25). Ritchie et al. noted that PAR2 expression was increased in human umbilical vein endothelial cells (HUVECs) following treatment with TNF-α or IL-1β. Subsequently, pro-inflammatory factors may induce an increase in PAR2, which may ultimately generate a positive feedback loop in inflammatory skin diseases (26).

#### 2.1.2 Impact of PAR2 on cell differentiation and skin barrier

Previous research has demonstrated that PAR2 is expressed above the basal layer in the human and mouse epidermis, and is most significantly expressed in the granular layer, suggesting that PAR2 may influence epidermal differentiation (27). Derian et al. reported that the activation of PAR2 inhibits the growth and differentiation of keratinocytes. Activation of PAR2 diminished protein expression of the differentiation marker transglutaminase-1 induced by either calcium or IFN-γ (28). Treatment with SLIGKV-NH2 has been shown to decrease the expression of markers of terminal differentiation in keratinocytes, including filaggrin, loricrin, and kallikrein 5 and 7 (29). Nevertheless, the influence of PAR2 on cell differentiation continues to be controversial. Demerjian et al. utilized caspase-14 and TUNEL(+) cells as corneocyte markers and concluded that PAR2 activation was indispensable for corneocyte terminal differentiation (30). Barr discovered that the PAR2 antagonist PZ-235 substantially inhibited the differentiation marker K10 in human keratinocytes (97). Shin et al. found that treated cultured immortalized keratinocyte cell line (SV-HEKs) with PAR2 agonist can increase loricrin and filaggrin expression, a terminal differentiation marker. They also noted that PAR2 expression in apocrine sweat glands is position-dependent. Strong PAR2 immunoreactivity was detected in the granular layer of normal human skin and the acrosyringium of eccrine sweat glands. In contrast, the granular layer of callused skin and the duct and gland cells of eccrine sweat glands exhibited faint PAR2 immunoreactivity, indicating that PAR2 is linked to the terminal differentiation of the epidermis and eccrine sweat glands (31). Lee et al. demonstrated that the small interfering RNA (siRNA)-mediated PAR2 knockdown in sebocytes led to aberrant differentiation and lipogenesis (32). The seemingly controversial effects of PAR2 on keratinocyte differentiation probably rely on the selection of differentiation markers, differences in experimental conditions and models, the complexity of signaling pathways and data analysis methods.

The primary function of the epidermis is to serve as a protective barrier against damage from the external environment and to prevent the leakage of water and electrolytes from the epidermis. Acute barrier disruption transiently elevates the ambient pH of normal SC from its usual acidic level (around 5.0) toward neutrality, subsequently activating serine proteases (SPs) in the outer epidermis. This increase in SP activity, in turn, activates PAR2, resulting in a delay in barrier recovery kinetics (30). Hachem et al. discovered that the permeability barrier’s recuperation is delayed and lamellar body (LB) secretion is inhibited by topical applications of the PAR2 agonist peptide SLIGRL. Conversely, PAR2 knockout mice exhibit accelerated barrier recovery and increased LB secretion, accompanied by enhanced lamellar body formation and elevated caveolin-1 expression. These results show that PAR2 is particularly important for the regulation of the permeability barrier and itch conduction (33). CAP1/Prss8 is a glycosyl-phosphatidylinositol-anchored serine protease mainly expressed in the granular and spinous layers of the epidermis and is crucial for epidermal barrier function. Frateschi et al. reported that transgenic expression of either CAP1/Prss8 (K14-CAP1/Prss8) or protease-activated receptor-2 (PAR2; Grhl3(PAR2/+)) in mouse skin leads to epidermal hyperplasia, ichthyosis, and itching. K14-CAP1/Prss8 ectopic expression impairs epidermal barrier function and causes skin inflammation. Surprisingly, the gross and functional manifestations of ectopic K14-CAP1/Prss8-induced phenotypes are wholly negated when they are introduced into a PAR2-null background, indicating that PAR2 is a critical mediator of epidermal barrier function (34).

The impact of PAR2 on barrier function has been confirmed by various clinical studies, the most notable being Netherton syndrome (NS). NS is a severe genetic skin disease with constant atopic manifestations that is caused by mutations in the serine protease inhibitor Kazal-type 5 (SPINK5) gene, which encodes the protease inhibitor lymphoepithelial Kazal-type–related inhibitor (LEKTI). The absence of LEKTI leads to the uncontrolled activity of kallikrein‐related peptidase 5 (KLK5), which activates KLK7, KLK14, and elastase 2 (ELA2). This premature degradation of desmosomes leads to the aberrant detachment of the stratum corneum from the granular layer (35, 36). Briot and colleagues provided that unregulated KLK5 directly activates PAR2 and induces NF-κB-mediated overexpression of TSLP, ICAM-1, TNFα, and IL-8, leading to increased permeability of the barrier and exacerbating skin inflammation and barrier defects (37). Briot et al. also generated Spink5/Par2 double knockout (DKO) mice. These mice display a dramatic decrease in TSLP expression, although stratum corneum detachment persists, confirming the role of the KLK5-PAR2 cascade in TSLP-mediated early proallergic signaling (38).

#### 2.1.3 PAR2 involvement in skin inflammatory response and immune response

Inflammation is a defensive response characterized by redness, swelling, heat, pain, and impaired function. Notably, PARs can actively modulate cellular signaling in a variety of inflammatory regions throughout the human body to facilitate or hinder inflammatory responses, suggesting a double-edged sword effect in inflammation (14, 39). For example, PAR2 activation was proven to increase intestinal wall thickness, granulocyte infiltration and bacterial translocation through the PAR2/Akt/mTOR pathway in inflammatory bowel disease (IBD) (40, 41). Whereas, blockage of PAR2 exacerbates inflammation in IBD co-occurring with metabolic syndrome partly through autophagy inhibition (42). This implies that PAR2 may have pleiotropic effects on inflammation depending on the environment.

**Figure 2:**
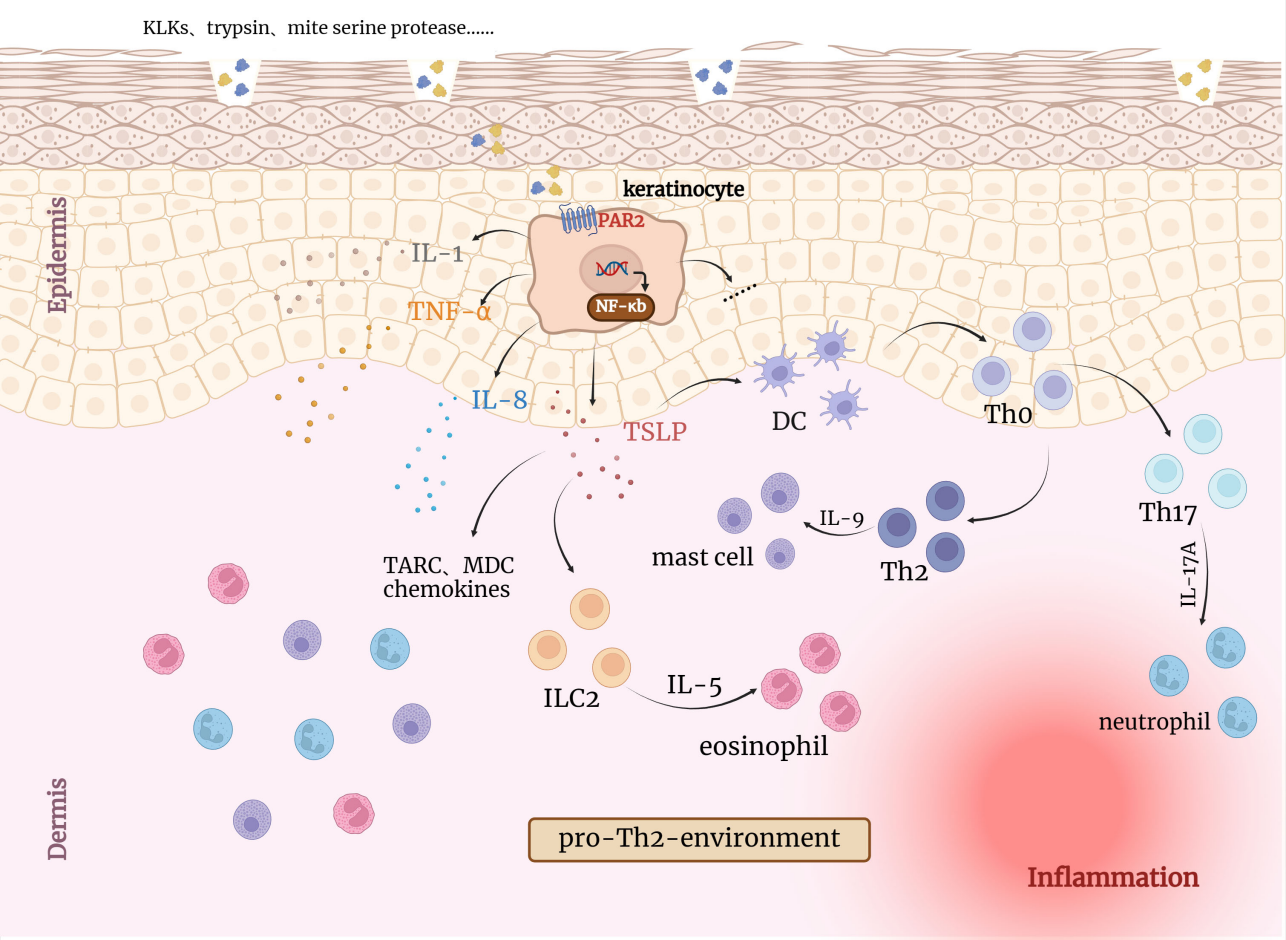
Activation of PAR2 by endogenous/exogenous serine proteases leads to inflammatory responses in the skin. KLKs, trypsin and other serine proteases can directly induce TSLP, IL8, IL1 and TNF-α overexpression through PAR2 and NF-κB pathway activation in keratinocytes, which can irritate inflammation responses. TSLP has been reported to activate resident LCs, which migrate to draining lymph nodes and promote the differentiation of naive T cells (Th0) into Th2 cells. Collectively, activated keratinocytes together with eosinophilic and mast cells induce pro-Th2 microenvironment favoring the development of an AD-like phenotype. DC, Dendritic cells; TSLP, Thymic stromal lymphopoietin; ILC2, type 2 innate lymphoid cell; TARC, thymic and activating regulatory chemokine; MDC, Macrophage-derived chemokine.

PAR2 can regulate cytokine responses, induce lymphocyte maturation and assist in the formation of immune organs (43). Thrombin efficiently cleaves PAR2 in a thrombomodulin(TM)-dependent manner, resulting in pro-inflammatory IL-8 release (44). It is worth mentioning that leukocyte migration, as one of the immune responses to inflammation, can be regulated by PAR2 through β-arrestin (45). PAR2 activation can increase vascular permeability and produce cytokines and adhesion molecules in the early phases of skin inflammation, resulting in further leukocyte aggregation (18, 19, 25, 46). Palmer et al. observed that activation of PAR2 on the surface of mast cells leads to degranulation and release of inflammatory mediators (47). Studies have shown that in human keratinocytes, PAR2 activation induces NF-κB activation, resulting in the production of pro-inflammatory cytokines and inducing Th2-associated inflammation (**Figure 2**) (13, 48). Moreover, wide-type mice had significantly higher levels of pro-inflammatory mediators than PAR2-KO mice during the acute contact dermatitis onset, along with leukocyte recruitment (44, 48, 49). PAR2 could contribute to skin immunity, by enabling ‘non-immune’ cells like keratinocytes to participate in skin protective functions. Sharlow and his colleagues observed that PAR2 activation increased the phagocytic capability of macrophages, fibroblasts and keratinocytes could be particularly significant to processes like wound healing or inflammation. This PAR2-mediated increase in phagocytic capability correlated with an increase in actin polymerization and α-actinin reorganization, cell surface morphological changes and increased soluble protease activity (50).

**Figure 3:**
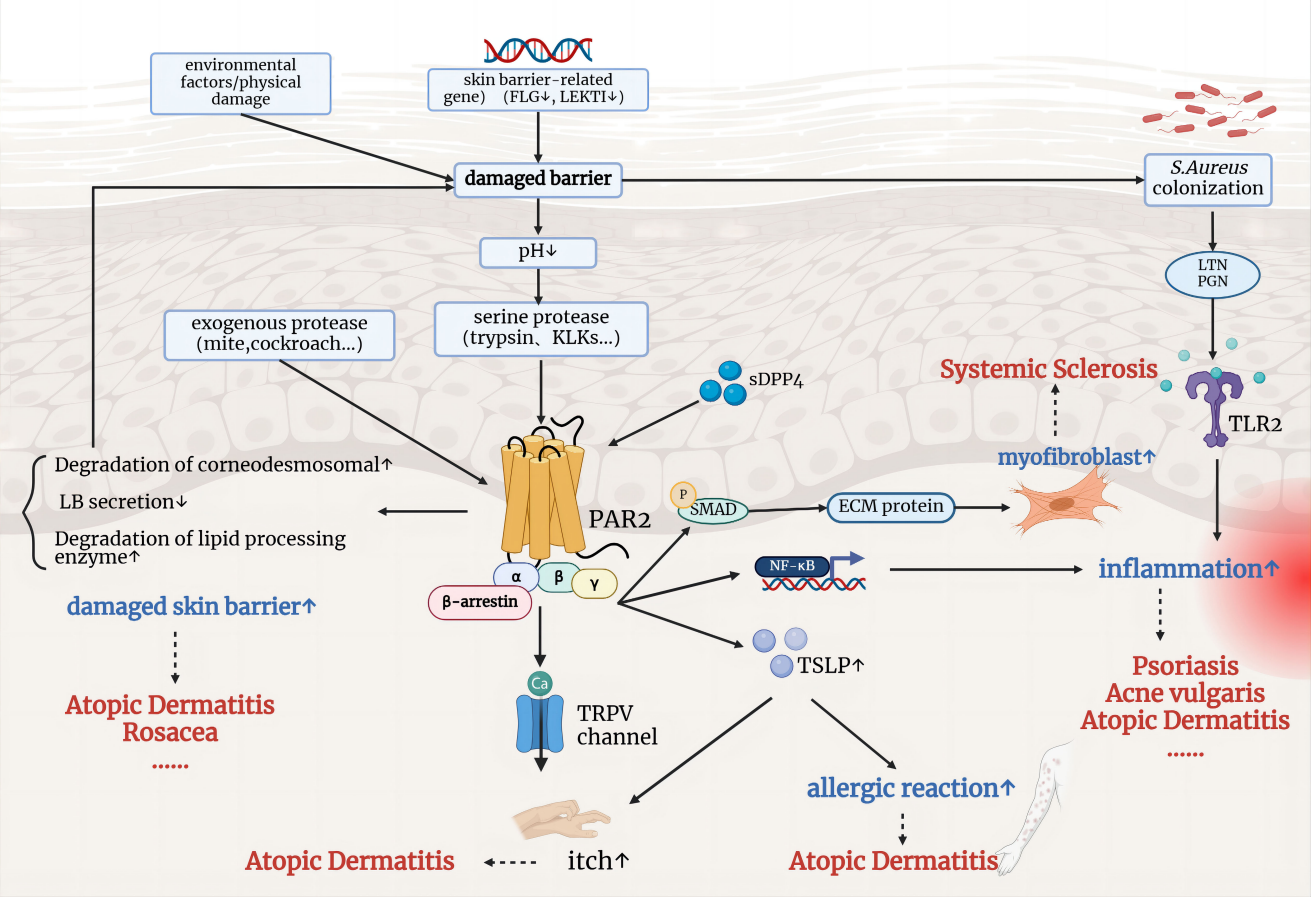
The involvement of PAR2 in the pathophysiological mechanisms of inflammatory skin diseases. Environmental factors and genetic mutations related to skin barrier function lead to a compromised barrier, allowing the entry of exogenous and serine proteases, which activate PAR2. Activated PAR2 contributes to further skin barrier damage, inflammation, and immune responses, and promotes itch through TRPV channels. These mechanisms are implicated in conditions such as atopic dermatitis, psoriasis, rosacea, and systemic sclerosis.

**Figure 3** illustrates the well-established impact of PAR2 on inflammatory skin diseases. Steinhoff et al. first observed positive PAR2 immunoreactivity in epidermal-dermal junction and perivascular areas in the skin of patients with AD and psoriasis, but not in normal skin (51). Giblin et al. generated a fully human PAR2 antibody specifically blocking the protease cleavage site in the N-terminal domain. The antibodies effectively inhibited PAR2-mediated intracellular calcium release and cytokine secretion in various cell types stimulated with trypsin. In a short-term mouse model of inflammation, the trans vivo DTH model, anti-PAR2 antibodies showed inhibition of the inflammatory swelling response, showing a 72% inhibition at a dose of 20 mg/kg (P<0.05), similar to the effect of dexamethasone at 10 mg/kg (52). These research findings indicate that PAR2 is involved in the inflammatory process *in vivo* and holds potential as a new therapeutic target for inflammatory skin conditions.

### 2.2 Role of PAR2 in inflammatory skin diseases

#### 2.2.1 PAR2, acne vulgaris and seborrheic dermatitis

Acne vulgaris is a chronic inflammatory disease of the pilosebaceous unit. *C. acnes* triggers acne inflammation by releasing various enzymes such as lipases, proteases, and hyaluronidases (53). It has been demonstrated that the extracellular proteinase produced by *C. acnes* is a heterogeneous mixture of at least three proteases, including a neutral protease containing a serine group and two alkaline proteases (54). Lee and colleagues found increased protease activity in acne lesions and enhanced PAR2 immunoreactivity in the vicinity of comedones. The culture supernatant of *C. acnes* activated calcium signaling in keratinocytes through PAR2 and upregulated the mRNA expression of several inflammatory mediators, including interleukin (IL)-1α, IL-8, tumor necrosis factor (TNF)-α, human beta-defensin (hBD)-2, LL-37, and matrix metalloproteinases (MMP)-1, -2, -3, -9, and -13. This effect was significantly reduced by the presence of a serine protease inhibitor and a selective PAR2 antagonist. These findings suggest that PAR2 is crucial in acne pathogenesis by triggering inflammatory responses to proteases secreted by *C. acnes (* 25).

Increased sebum content and altered composition are also important factors contributing to the development and progression of acne. PAR2 expression was detected at both the mRNA and protein levels in SZ95 sebocytes. Detection of intracellular Ca^2+^ mobilization by the *C. acnes* culture supernatant suggests that it is a potent activator of PAR2 on sebocytes. The mRNA levels of SREBP1 in sebocytes were elevated with the treatment of PAR2 agonist peptide(AP), and the protein levels of both precursor and mature forms of SREBP-1 increased as well. These findings suggest that PAR2 is involved in the differentiation and production of sebum in sebocytes. Moreover, PAR2 AP induced IL-8, TNF-a and hBD-2 transcription in sebocytes (32). These findings indicate that PAR2 probably plays a crucial role in the development and progression of acne, as it mediates differentiation, inflammation, and innate immunity in SZ95 sebocytes in response to *C. acnes*.

Antibiotics such as macrolides and tetracyclines are frequently employed to treat acne. Their therapeutic effects are not only related to antibacterial properties but also anti-inflammatory activity and immunomodulatory effects. Ishikawa et al. found that tetracycline and its derivatives, such as doxycycline and minocycline, significantly inhibited IL-8 release in normal human epidermal keratinocytes (NHEKs) triggered by the PAR2 agonist peptide SLIGKIV-NH2. This research suggests that tetracyclines can effectively reduce cutaneous inflammation by attenuating the PAR2/IL-8 axis in keratinocytes (55). Another study showed that doxycycline indirectly inhibits the production of KLKs and TSLP by reducing the secretion of MMPs in keratinocytes. This pathway may also be implicated in the anti-inflammatory process of doxycycline, as KLK5 and KLK14 have the ability to activate PAR2. Similarly, studies have found that macrolide antibiotics can modulate inflammatory response by attenuating the PAR2/IL-8 axis (56). Seborrheic dermatitis(SD) is also a skin disorder characterized by inflammatory scaling rush in seborrheic areas of the body. Viodé et al. demonstrated an increase in cathepsin S, PAR2 and histamine protein levels in SD lesions, indicating cathepsin S may be implicated in the pathogenesis of seborrheic dermatitis by activating PAR2 (57).

#### 2.2.2 PAR2 and rosacea

Rosacea is a chronic inflammatory skin disorder, characterized by facial flushing, telangiectasia, inflammatory papules and pustules on the central region of the face (58). Previous studies have reported that serine proteases (SPs) activity and antimicrobial peptides (AMPs) expression are significantly elevated in the skin of rosacea patients (59). The epidermal antimicrobial peptide is degraded by SPs to its active form, cathelicidin LL-37. The latter can mediate vascular action and inflammation, which are features of rosacea. AMPs are important defenders in the innate immune response of the skin and can directly kill invading microorganisms as well as trigger the adaptive immune response, suggesting a potential therapeutic target for rosacea (60). Furthermore, cathelicidin LL-37 and PAR2 show significant positional correlation on immunohistochemistry. Cathelicidin LL-37, PAR2, and VEGF mRNA and protein levels were all elevated after the administration of a PAR2 agonist peptide to keratinocytes (61).

Zhong et al. conducted a two-week randomized, vehicle-controlled, split-face study on 30 rosacea patients using 3% tranexamic acid solution and vehicle-control treatment. Tranexamic acid, acting as a serine protease inhibitor, has demonstrated efficacy in hastening the repair of compromised skin barriers. The nuclei and cytoplasm of the normal epidermis exhibited a mild expression of PAR2, whereas rosacea patients exhibited a more intense staining of PAR2 in both the nuclei and cytoplasm. Tranexamic acid inhibits calcium mobilization induced by PAR2 activation, improves permeability barrier function and relieves clinical manifestations of rosacea patients (62). After 4-6 weeks of topical application of 10% tranexamic acid, Jakhar et al. observed a significant reduction in facial erythema, telangiectasia, and stinging sensation in patients with rosacea (63). We hypothesize that the pathogenesis of rosacea may be influenced by exogenous stimuli (such as heat, spicy food, UV, physical, and microbes) and serine proteases (such as kallikrein 5 and kallikrein 14) that upregulate cathelicidin LL-37 through enhanced PAR2 signaling. This, in turn, modulates innate immune responses and vascular activity.

#### 2.2.3 PAR2 and psoriasis

Psoriasis is a chronic autoimmune skin disease characterized by red, itchy, and scaly skin patches (64). Research has demonstrated that PAR2 expression is elevated in the epidermis of psoriasis patients, and it may contribute to the pathogenesis of psoriasis by raising TNF-α (65). Moreover, the pathogenesis of psoriasis has been linked to the production of IL-8 by keratinocytes. A novel serine protease, human airway trypsin-like protease (HAT), was isolated from mucoid sputa of patients with chronic airway diseases. Iwakiri et al. detected that the immunofluorescence staining of HAT in psoriatic epidermis was more extensive and intense than that in normal skin. HAT treatment resulted in a synergistic increase in IL-8 release, while PAR-2 inhibition led to a decrease in IL-8 release induced by HAT (66). Therefore, HAT may facilitate the accumulation of inflammatory cells in the epidermal layer of psoriasis by promoting PAR2-mediated IL-8 production, thereby contributing to the aggregation of inflammatory cells in the psoriatic epidermis.

Carvalho et al. observed a substantial increase in the percentage of mast cells expressing PAR2 in the lesional skin of psoriasis compared to the non-lesional skin when analyzing the expression of PAR2 immunoreactivity in mast cells isolated from lesional and non-lesional skin of patients with plaque psoriasis. Activation of cord-blood-derived mast cells (CBMC) with the PAR2 agonist peptide (2-furoyl-LIGRLO-NH2) led to an increase in the secretion of IL-8, but no histamine release was observed (67). Abji et al. isolated tryptase-6, a serine protease that can activate PAR2, from the synovial fluid of patients with psoriatic arthritis (PsA) and observed increased levels of monocyte chemoattractant protein-1 (MCP-1) from PsA patient-derived monocytes/macrophages in response to PAR2 activation, highlighting a potential mechanism of PAR2-mediated recruitment of monocytes/macrophages to the PsA joint (68). In the murine psoriasis-like epidermis, KLK6, KLK7, and KLK8 were discovered to be overexpressed (69). Since PAR2 is a downstream target of KLKs, we hypothesized that overexpressed KLKs lead to inflammation and aberrant differentiation of keratinocytes in psoriasis occurrence.

#### 2.2.4 PAR2 and atopic dermatitis

Atopic dermatitis (AD) is a chronic pruritic skin disease characterized by skin hyperreactivity, recurrent erythema, itching, and eczema-like lesions (70). PAR2 expression is significantly increased by keratinocytes throughout the epidermis in skin lesions of patients with AD and expresses at lower levels in normal skin (51). PAR2 is implicated in the development and occurrence of AD, as evidenced by the detection of KLK5, -7, -8, and -14 in the perspiration of AD patients (79). Epidermal serine protease activity is increased in the skin lesions of AD patients due to the overexpression of KLKs, which facilitates the invasion of microorganisms and allergens and further aggravates AD (24, 71). Furthermore, Briot and colleagues have previously reported that unregulated KLK5 directly activates PAR2 and induces NF-kB-mediated overexpression of TSLP, ICAM-1, TNF-α, and IL-8 (37). TSLP is instrumental in the development of AD by promoting the production of Th2 cell-promoting cytokines by activated mast cells in conjunction with TNF-α (72).

Growing evidence indicates that PAR2 is intimately involved in driving itch during atopic dermatitis. Proteases that activate PAR2 in primary sensory neurons stimulate the release of substance P and Calcitonin Gene-Related Peptide (CGRP) in peripheral tissues, leading to neurogenic inflammation and pruritus (73). TRPV3, a warm temperature-sensitive Ca^2+^-permeable cation channel that is abundantly expressed in cutaneous keratinocytes, has been confirmed to play a critical role in the transmission of PAR2-induced itch. Scratching would further activate the PAR2-TRPV3 pathway in the epidermis, leading to the release of TSLP and the exacerbation of the vicious itch-scratch cycle (74). Moreover, antihistamines lack efficacy in treating itch in AD, suggesting the existence of histamine-independent itch pathways in AD (73).

Transgenic mouse models as well as human studies have demonstrated that a strong association exists between PAR2 overexpression and AD. Frateschi et al. found that keratinocyte-specific overexpression of PAR2 results in AD-like lesions characterized by epidermal hyperplasia, ichthyosis, and itching (22). With the application of house dust mites, Meng et al. observed that Grhl3PAR2^/+^ mice exhibit atopic-like inflammation, scaly dry skin, epidermal hyperplasia, pruritus behaviors, and increased brain natriuretic peptide transcription in adjacent sensory neurons (75). Buhl et al. established a murine model with epidermal overexpression of PAR2 (PAR2OE) and found that PAR2 is sufficient to drive barrier dysfunction, inflammation, and pruritus, highlighting the importance of serine proteases and PAR2 in AD pathogenesis (76).

Thus, PAR2 antagonism and/or selective protease inhibitors may represent a novel approach for the treatment of AD.Tsujii et al. investigated the involvement of tryptase and proteinase-activated receptor (PAR) subtypes in spontaneous scratching in NC mice. Intravenous administration of an anti-PAR2 antibody significantly reduced spontaneous scratching in mice with dermatitis. Conversely, intradermal injection of the PAR2-activating peptide SLIGRL-NH2 induced scratching behavior in healthy mice, whereas peptides activating PAR1, PAR3 or PAR4 did not produce the same effect (77). IL-27 is a member of the IL-6/IL-12 cytokine family, a group that also includes IL-31, a critical mediator in itch generation and cutaneous AD-associated pruritus. Li et al. discovered that the pruritus response in the cheek model was exacerbated by coinjection SLIGRL or trypsin and IL-27, which in turn increased the expression of Tslp via PAR2 (78). Passive cutaneous anaphylaxis-induced scratching behavior was substantially inhibited by pretreatment of the ICR animals with PAR2-neutralizing antibody and protease inhibitor (leupeptin) (79).

#### 2.2.5 PAR2 and systemic sclerosis

Systemic sclerosis (SSc), also called scleroderma, is an immune-mediated rheumatic disease that is characterized by fibrosis of the skin and internal organs and vasculopathy. Fibroblasts are instrumental in the pathophysiology of fibrotic diseases by coordinating the remodeling of the extracellular matrix (80). Cevikbas et al. observed PAR2 immunoreactivity in fibroblasts of SSc skin, but not in healthy skin. Additionally, PAR2 co-localized with 21 ± 8% a-SMA-positive myofibroblasts. In human dermal cultured fibroblasts (HDF), PAR2-induced intracellular calcium mobilization was only measurable after bleomycin pretreatment, indicating PAR2 may be inducible and upregulated by an unknown stimulus in systemic SSc (81). Dipeptidyl peptidase-4 (DPP4) is a functional requirement for fibroblast activation and tissue fibrosis and may serve as an activation marker (82). The signaling signature induced by sDPP4 suggests that sDPP4 might be an agonist for PAR2. PAR2 silencing prevents sDPP4-induced ERK1/2 activation and NF-κB phosphorylation, as well as IL-6 and IL-8 upregulation in human smooth muscle cells (83). Lee et al. demonstrated that the PAR2 antagonist or knocking down PAR2 can prevent sDPP4–induced fibrotic marker expression via SMAD/NF-κb signaling in fibroblasts. This implies that PAR2 is a receptor for soluble DPP4 and facilitates the development of fibrosis (84).

## 3 Potential targeted therapies based on PAR2

PAR2 is a significant target for therapeutic intervention due to its significant function in the development of inflammatory diseases. Small molecules, antibodies, inhibitory peptides, peptidomimetics, and cell-penetrable pepducins have all been developed in recent years as PAR2 modulators (85). The representative modulators for each type are listed in **Table 2**. In this section, we concentrate on the function of PAR2 modulators in inflammatory skin diseases.

**Table 2:** PAR2 modulators, mechanism, and potential applications.

PAR2 modulators	Mechanism	Potential applications	ref
Peptides			
FSLLRY-NH2	↓TRPV1, TRPA1 receptor expression and substance P, CGRP in SCI-rat model ↓IL-8, IL-1β, TNF-α via SAPK/JNK in HepG2 cells ↓calcium release and immune response in bronchial/tracheal cells	1.alleviated neuropathic pain and thermal hyperalgesia in SCI mouse model 2.used as asthma adjuvant therapy	(96, 97) (98), (99)
Peptidomimetics			
K-14585 K-12940	↓NF-kB transcriptional activity in primary human keratinocytes ↓SLIGKV-induced Ca^2+^ mobilization and IL-8 production in primary human keratinocytes * K-14585 (at 30 mM) was found to activate p38 MAP kinase, NFκB and IL-8 pathways	1.lowered plasma extravasation in the dorsal skin of guinea pigs and reduced salivation in mice 2.inhibited the relaxation of rat-isolated aorta induced by SLIGRL-NH2	(86) (100),
C391 C781	↓calcium release and β-arrestin/MAPK signaling in 16HBE14o-cell and HEK 293 cells. ↓mechanical and spontaneous nociceptive behaviors in response to small molecule PAR2 agonists	1.attenuated inflammation, mucus production, mucus cell hyperplasia, and AHR in acute allergen-challenged murine models 2.blocked certain aspects of signaling of protease-evoked pain in mice	(87) (88, 101)
Pepducins			
P2pal-18S	↓SLIGRL-NH2 induced calcium transients and neutrophil migration ↓GM-CSF production, T-cell activation and macrophage polarization in splenocytes	1.alleviated biliary pancreatitis severity 2.reduced SLIGRL-induced paw edema in mouse models 3.potential treatment for MS and neuroinflammatory diseases	(89, 102, 103)
PZ235	↓IL-1β, MCP-1, macrophage accumulation and MMP2 expression in the abdominal aorta ↓fibrosis, necrosis, ROS and inflammation in liver ↓NF-κB, TSLP, TNF-α, and differentiation marker K10 in human keratinocytes and suppressed IL-4 and IL-13 in mast cells.	1.reduced AAA progression, liver fibrosis and hepatocellular necrosis in a mouse model 2. potential treatment for AD and itch	(90, 104) (91),
Non-peptide small molecules			
ENMD-1068	↓calcium release(dose-dependently) ↓eosinophil and neutrophil counts, CXCL1, CCL5, amphiregulin, and IL-6 and 13 levels, ↑IL-10 level	1.inhibited C/K-induced joint inflammation in murine arthritis models 2.alleviated allergen-mediated acute lung inflammation in OVA-treated mice	(105, 106)
GB83 GB88	↓calcium release and PAR2-mediated p65 NFκB phosphorylation ↓the mRNA expression of IL-6 and IL-1β ↓oxidative stress and inflammatory response in hairless mice ↓soluble DPP4–induced expression of fibrotic proteins in fibroblasts. *GB83 was further proved to be an agonist of PAR2.	1.attenuated inflammatory response in acute rat paw edema 2.prevented skin photoaging and skin fibrosis	(95) (84, 94),
AZ3451 AZ8838 AZ7188	↓calcium release and β-arrestin recruitment ↓P38/MAPK, NF-κB and PI3K/AKT/mTOR pathways induced by IL-1β in chondrocytes ↓ox-LDL-induced expression and production of IL-6, TNF-α,IL-8 MMP9, MMP2 and ICAM-1 via NF-κb pathway	1.alleviated the surgery-induced cartilage degradation in rat OA model 2. attenuated tubular dilation, tubulointerstitial and hyperuricemia-induced inflammation	(107–110)
I-287 I-191	↓IL-8 release in HCT116 and A549 cells ↓tryptase-induced cellular migration and release of GH in human bronchial epithelial cells	1.reduces CFA-induced inflammation in mice 2.blocked tryptase-enhanced epithelial wound healing	(111) (112)
Antibodies			
SAM11	↓inhibited Th2 inflammation (decreased levels of IL-4, IL-5, and CCL11) ↓inhibited Th17 responses and the levels of chemotactic molecules for neutrophils and other immune cells	1.abrogated collagen-induced arthritis model 2.prevented inflammation, AHR, and collagen accumulation in a long-term model of cockroach-dependent allergic airway inflammation	(105) (113)
MEDI0618	unpublished	unpublished	(104)

*CGRP, Calcitonin gene-related peptide; SCI, spinal cord injury; AHR, airway hyperresponsiveness; MS, Multiple Sclerosis; ROS, reactive oxygen species; AAA, abdominal aortic aneurysm; AD, atopic dermatitis; C/K, carrageenan/kaolin; OVA, ovalbumin; LDL, low-density lipoprotein; OA, Osteoarthritis; GH, growth hormone; CFA, Complete Freund’s Adjuvant. ↑ represents for up-regulated:↓ represents for down-regulated.

In 2009, Plevin and colleagues first developed PAR2 peptide antagonists K14585 and K12940. These peptides demonstrated competitive inhibition of PAR2 binding and substantially reduced PAR2-calcium fluxes in primary human keratinocytes. Among these two compounds, K14585 can significantly reduce plasma extravasation in guinea pig back skin and inhibit inflammatory response (86). Kume et al. found C781, a β-arrestin-biased PAR2 antagonist effectively prevented and reversed mechanical and spontaneous nociceptive behaviors in response to PAR2 agonists, mast cell activators and neutrophil elastase. This finding suggests that by blocking specific aspects of signaling by the receptor, protease-evoked pain can be effectively blocked (87). In an acute allergen-challenged mouse model, Schiff et al. demonstrated that C781 modulated airway hyperresponsiveness, airway inflammation, and mucus overproduction in the small airways (88).

Pepducins are small lipidated peptide fragments designed from the amino acid sequences of the intracellular loop (ICL) domains of GPCRs. Sevigny and colleagues reported the development of first-in-class cell-penetrating lipopeptide pepducin antagonists of PAR2, P2pal-18S. They confirmed P2pal-18S was highly efficacious in blocking PAR2-dependent inflammatory responses in mouse models of inflammatory paw edema (89). Further developments in the PAR2 pepducin series have led to PZ-235, with blockade of PAR2 G protein signaling through the pepducin aligning with ICL3 and TM6, which is currently being explored as a potential therapeutic candidate for liver fibrosis and atopic dermatitis (90, 91). Barr et al. reported that PZ-235 significantly inhibited PAR2-mediated expression of inflammatory factors NF-κB, TSLP, TNF-α, and differentiation marker K10 in human keratinocytes and suppressed IL-4 and IL-13 in mast cells. PZ-235 significantly reduced itching caused by degranulation of mast cells in mice (91). These findings indicate that PZ-235 has the potential to provide extensive therapeutic benefits as a disease-modifying treatment for atopic dermatitis and pruritus.

A breakthrough in the search for PAR2 antagonists was the development of the GB series of non-peptide antagonists, as it demonstrated a higher bioavailability. Modifications of the C-terminus gave rise to the compounds GB83 and GB88 (92). Inhibition of PAR2 activation using GB83 decreased the inflammatory response and oxidative stress and decreased NF-κB/FoxO6 phosphorylation during skin photoaging (93). However, it was further proved to be an agonist of PAR2 (94). Suen et al. found that GB88 was orally active and anti-inflammatory *in vivo*, inhibiting acute rat paw edema elicited by PAR2 agonists and proteolytic of PAR2 (95).

Another approach to developing PAR2 inhibitors is through humanized blocking antibodies. Glibin et al. generated fully human antibodies that specifically block the N-terminal protease cleavage site of PAR2, effectively inhibiting PAR2-mediated intracellular calcium release and cytokine secretion in a variety of cells stimulated by trypsin. Pharmacodynamic studies showed that anti-PAR2 antibody has a significant inhibitory effect on the murine delayed-type hypersensitivity(DHT) model of inflammatory swelling responses (52).

Currently, research targeting PAR2 for therapeutic purposes mainly focuses on directly intervening in PAR2 activation. With the resolution of crystal structures and novel agonist-bound models, non-peptide small molecules may offer a promising future in PAR2-targeted therapy. Compared with conventional ligand-receptor interactions at the cell surface, PAR2-targeted therapy is hampered by challenges. (i) Tethered ligands can be activated by a variety of proteases, such as tryptase, kallikreins, and specific cathepsins. Targeting a single protease might not completely block PAR2 activation, potentially leading to unintended effects mediated by other proteases. (ii) PAR2 is widely distributed throughout the body. Different cell types may respond differently to PAR2 modulators, potentially causing off-target effects in non-target tissues. (iii) PAR2 belongs to the G-protein-coupled receptor (GPCR) family, with many GPCRs sharing similar structures and functions. PAR2 modulators might cross-react with other GPCRs, resulting in non-specific effects. (iv) Drug safety is also a matter that must be taken into account. PAR2 plays a crucial role in inflammatory responses. Activation or inhibition of PAR2 may lead to excessive inflammation or suppression of normal immune functions, increasing the risk of infections or other diseases. It is imperative to identify and employ biomarkers to evaluate patients who are most likely to benefit from PAR2-targeted therapies. Large-scale, comprehensive, and multi-center clinical trials should be conducted to thoroughly assess the safety and efficacy of PAR2 modulators, and monitor potential safety issues through long-term follow-up studies. The initial humanized monoclonal PAR2 antibody has advanced to phase I clinical trials (NCT04198558) for the treatment of chronic pain (104). Although the trial outcomes are still unknown, this advancement represents a significant step toward bringing PAR2 therapies closer to clinical application.

## 4 Conclusions and future perspectives

PAR2 plays a multifaceted role in skin inflammatory diseases, impacting various aspects of inflammation, immune responses, skin barrier repair, and tissue remodeling. Analyzing and understanding its underlying molecular mechanisms is critical for figuring out disease etiology and developing therapeutic interventions. The role of PAR2 in inflammatory skin diseases has not been completely elucidated due to its widespread distribution. Further investigation is required to determine the relationship between PAR2 and other inflammatory skin diseases, including urticaria and pemphigus. New strategies for managing inflammatory skin diseases are anticipated to be revealed. Critical considerations in the development and application of PAR2 modulators include potential off-target effects and safety concerns. By developing tissue-specific PAR2 modulators, applying biomarkers, and conducting comprehensive clinical trials, these issues can be mitigated, thereby optimizing treatment outcomes and offering patients safer and more effective therapeutic options.
